# Polyhydroxybutyrate production by a sugarcane growth promoter bacterium

**DOI:** 10.1186/1753-6561-8-S4-P246

**Published:** 2014-10-01

**Authors:** Alexander Machado Cardoso, Carlos Vinícius Ferreira da Silva, Aleksander S de Paula Silva, Vânia L Muniz Pádua

**Affiliations:** 1 Fundação Centro Universitário Estadual da Zona Oeste (UEZO), Rio de Janeiro, Brazil

## Background

The plastics derived from petrochemicals have many applications. However, public concern and environmental laws led to conservation policies and establish programs that stimulate the research and use of new products based on renewable resources. Polyhydroxyalkanoates (PHAs) are biopolymers synthesized by microorganisms under conditions of nutrient stress with similar properties to conventional plastics and are completely biodegradable. One of the PHAs is polyhydroxybutyrate (PHB), which accumulated in several bacteria [1]. *Gluconacetobacter diazotrophicus *is a plant-growth-promoting bacterium, which interacts and lives within sugarcane plants [2]. Genome annotation of this bacterium offers a new scenario to better understand plant-bacteria interactions and reveals novel potential bioproducts. Sugarcane is cultivated mostly for sugar and fuel ethanol production. In this work, we propose the production of biodegradable plastics from sugarcane coupled to its natural partner growth promoter bacterium, *G. diazotrophicus*.

## Methods

An ORF encoding a conserved hypothetical protein sharing approximately 53% identity with the PHB synthase from *Acidiphilium multivorum *was identified within *G. diazotrophicus *genomic sequence. This putative *G. diazotrophicus *PHB synthase gene, consisted of a 987 bp (encoding 328 residues), with a molecular mass of approximately 35 kDa. This PHB synthase was analyzed for structural motifs by scanning against PROSITE patterns and profiles (http://www.expasy.org/prosite) and against Pfam (http://pfam.sanger.ac.uk) to certify about the presence of domains associated with PHB. A multiple alignment analysis was performed using CLUSTALW and the phylogenetic trees were generated using MEGA based on the maximum likelihood (ML) method and the topology of the trees was evaluated by bootstrap analysis on the basis of 1,000 replications.

## Results and conclusions

Analysis of the *G. diazotrophicus *genome sequence for putative PHB polymerase gene resulted in the identification of a protein with a poly(R)-hydroxyalkanoic acid (PHA) synthase domain, class III, that represents the PhaC subunit of a heterodimeric form of the polyhydroxyalkanoic acid synthase, which links D-(-)-3-hydroxybutyrl-CoA to an existing PHA molecule by the formation of an ester bond. A deeper analysis revealed that *G. diazotrophicus *PHB polymerase and a putative polyhydroxyalkanoate synthesis repressor (PhaR) are clustered with hypothetical proteins, a hydrolase and an acetoacetyl-CoA reductase, suggesting they are gathered functionally, maybe as operon. The PHA synthase appear to require PhaR for activity *in vivo *and *in vitro *[3]. This PhaR exhibts the PHB/PHA accumulation regulator DNA-binding domain, that binds short chain hydroxyalkanoic acids and PHA granules, and may regulate the expression of itself, of the phasins that coat granules, and enzymes that direct carbon flux into polymers stored in granules [4]. Interesting, the phylogenetic analysis revealed that *G. diazotrophicus *PHB forms a cluster with other relevant PHB enzymes with intriguing metabolic activities. Although additional approaches are still needed for reach production levels, these data show that *G. diazotrophicus *has potential for producing PHB and be employed in the bioplastic production, integrated to the sugarcane industry.
